# Correlates of left ventricular mass in hypertensive Nigerians: an echocardiographic study

**Published:** 2010-04

**Authors:** Okechukwu S Ogah, Afolabi E Bamgboye

**Affiliations:** Department of Medicine, Federal Medical Centre, Idi-Aba, Abeokuta, Nigeria; Departments of Epidemiology, Medical Statistics and Environmental Health (EMSEH), College of Medicine, University of Ibadan, Nigeria

**Keywords:** hypertension, left ventricular mass, correlates, determinants, Nigeria

## Abstract

**Background:**

Studies have shown that left ventricular mass, diagnosed by echocardiography, correlated poorly with blood pressure, even when the 24-hour ambulatory blood pressure monitoring was taken into account in the analysis. This may be partly because there are other determinants of left ventricular mass such as age, gender, neurohormonal factors and heredity.

Knowledge of the correlates of left ventricular mass could help design individual and population strategies to prevent or reverse left ventricular hypertrophy. To the best of our knowledge, there is a paucity of such studies in native Africans. Hence the purpose of this study was to define the correlates of left ventricular mass in hypertensive Nigerians.

**Methods:**

The study was a retrospective analysis of prospectively collected data in 285 hypertensive subjects. Echocardiographic left ventricular mass was determined using the standard formula. Stepwise multiple regression analysis was used to determine the independent predictors of left ventricular mass with a probability value to enter and remove of *p* < 0.05.

**Results:**

There were 153 men (53.7%) and 132 women (46.3%) in the study. The mean age of all subjects was 58.2 ± 13.7 years. There was no significant gender difference in most of the echocardiographic parameters. In a stepwise multiple regression analysis, left ventricular wall tension, left ventricular wall stress, left atrial size, diastolic blood pressure, alcohol consumption and a family history of hypertension were the independent predictors of left ventricular mass in this population. The optimum multivariate linear regression main effects had an adjusted model, *r*^2^ of 0.945, thus explaining about 95% of left ventricular mass variability.

**Conclusion:**

Mechanical or haemodynamic factors possibly interacting with genetic and social factors are the likely determinants of left ventricular mass in hypertensive Nigerians. Therefore modulation of some of these factors pharmacologically or non-pharmacologically will be of benefit in the management of this patient population.

## Summary

Left ventricular hypertrophy (LVH) has been shown to be a significant risk factor for adverse outcomes both in patients with hypertension and in the general population.^1^,^2^ Although for many years LVH was thought to be a beneficial compensatory mechanism for maintaining wall stress in left ventricular (LV) pressure and volume overloads, epidemiological studies using electrocardiography (ECG) and more recently echocardiography (ECHO) have elucidated the profound independent risk of LVH for congestive heart failure (CHF),^3^,^4^ coronary artery disease (CAD),^5^,^6^ life-threatening arrhythmias^7^ and cardiac mortality.^1^-^4^

Studies have shown that, among other things, increased left ventricular mass (LVM) is associated with a significant excess of cardiovascular morbidity and mortality.^8^ This is independent of the co-existence of coronary artery disease and hypertension.^8^ There is a threefold increase in the mortality rate in persons with either of these conditions in addition to increased LVM.^8^ The risks of coronary, peripheral or cerebral vascular disease are also raised even among normotensive individuals with LVH.^1^,^3^,^4^

LVH defined by ECG has been shown to be an independent risk factor for cardiovascular events.^4^ More recently LVH defined by echocardiography, a more sensitive diagnostic tool, has similarly been shown to increase cardiac risk.^9^ Recent data from the LIFE study (Losartan Intervention For Endpoint reduction in hypertension) revealed a close correlation between microalbuminuria and ECG-determined LVH.^10^-^12^

Regression of LVH has also been shown to be possible using either antihypertensive drugs (except hydralazine and minoxidil) or non-pharmacological lifestyle modifications. Therefore an understanding of the factors promoting increased LVM in hypertensive Nigerians may permit the development of strategies aimed primarily at preventing LVH or promoting regression more effectively. Moreover, to the best of our knowledge, no work of this nature has been carried out in this environment.

The objectives of the present study were to assess the nonhaemodynamic and haemodynamic correlates of LVM among hypertensive Nigerians seen at the University College Hospital (UCH), Ibadan, Nigeria.

## Methods

The study was carried out at the Medical Outpatient Unit of the University College Hospital, Nigeria. It was a retrospective analysis of prospectively collected data. Eligible patients were men and women aged 18 years and older with established hypertension, who were attending the hypertension clinic at the hospital. Diagnosis of hypertension was based on systolic blood pressure of ≥ 140 mmHg and diastolic blood pressure of ≥ 90 mmHg or those on antihypertensive therapy.^13^

Subjects with a previous history or symptoms of ischaemic heart disease, echocardiographic evidence of regional wall motion abnormalities, or established congestive heart failure were excluded from the study. Other exclusion criteria included patients with hypertrophic cardiomyopathy, left bundle branch block and those with incomplete echocardiography reports. Ethical approval was obtained from the institution’s ethical review board.

The estimated minimum sample size was 255 hypertensive subjects. The calculation of the minimum sample size was based on the prevalence of echocardiographic LVH in hypertensive subjects at the UCH, Ibadan, which was between 16.5 and 25.8% depending on the cut-off value used in defining LVH.^14^ The study power was 80% (20% beta error) and the alpha error was set at 5% (0.05).

## Clinical evaluation

Baseline clinical and demographic characteristics were obtained from the subjects’ case notes. These included date of birth (age), gender, history of diabetes, history of smoking, duration of their hypertension, and family history of hypertension. Also obtained were the blood pressure, pulse rate, body weight and height at the time of echocardiography. Body mass index (BMI) was calculated using the formula:

BMI (kg/m^2^) = $\frac{weight}{height²}$

Body surface area (BSA) was calculated using the formula of Dubois:^15^

BSA (m^2^) = 0.0001 × 71.84 × [weight (kg)]^0.425^ × [height (cm)]^0.725^

## Echocardiography

All the echocardiograms were performed with the use of a commercially available echo-machine (ALOKA SSD-1, 700) and a 3.5-MHz linear array transducer. This was performed on each subject in the left lateral decubitus position. All measurements were made according to the American Society of Echocardiography leading edge-to-leading edge criteria.^16^

LV measurement was obtained at end-diastole and end-systole. The LV measurements recorded included interventricular septal thickness at end-diastole (IVSTd), posterior wall thickness at end-diastole (PWTd), and the LV internal dimensions at end-systole (LVIDs) and end-diastole (LVIDd). Other parameters obtained were left atrial diameter, aortic root diameter, indices of LV diastolic function [early-filling velocity (E-velocity), late-filling velocity (A-velocity) and deceleration time (DT)].

Two experienced cardiologists performed the echocardiography. In our laboratory, the intra-observer concordance correlation coefficient ranged from 0.76 to 0.98 while that of the inter-observer concordance ranged from 0.82 to 0.96.^17^

## Calculation of echocardiographically derived variables

Left ventricular mass was calculated using the formula that has been shown to yield values closely related (*r* = 0.90) to necropsy LV weight and which has good inter-study reproducibility.^18^

LVM (ASE) = 0.8 [1.04 (IVSTd + LVIDd + PWTd) 3 + 0.6 g

Relative wall thickness was calculated as twice the posterior wall thickness/LV internal dimension in diastole. Relative wall thickness of 0.43 or greater was considered abnormal.^19^ LV hypertrophy was considered present when LV mass exceeded 51 g/m^2.7^ in both men and women.

## Haemodynamics

Left ventricular volumes were estimated using the formula of Teichholz *et al*.^20^ These include: LV end-diastolic volume, LV-end systolic volume, stroke volume and cardiac output. These measurements have been validated with the invasive Doppler echocardiographic method.^21^

LV end-diastolic volume (EDV) (ml) = 7[LV internal in diastole (LVIDd) + 2.4] × LVIDd

LV end-systolic volume (ESV) (ml) = 7[LV internal in systole (LVIDs) + 2.4] × LVIDs

Stroke volume (SV) (ml) = EDV – ESV

Cardiac output (CO) (ml) = SV × heart rate (HR)

Mean arterial blood pressure (MAP) (mmHg) was calculated as: diastolic BP + 1/3 pulse pressure

Total peripheral resistance was assessed as: $\frac{MAP\;\times 80}{CO}$

Pulse pressure (mmHg) was estimated as: SBP – DBP

Fractional shortening (%) = $\frac{100\;\times (LViDd\;-LVIDs)}{LViDd}$

Ejection fraction (%) = $\frac{100\;\times (EDV-ESV}{EDV}$

The ratio of pulse pressure to stroke volume was used as an indirect estimate of global arterial stiffness.^22^,^23^

LV peak wall stress (dynes/cm^2^) was calculated as: $\frac{0.86\;\times (0.0334\;\times SBP\;\times LViDd)}{\left(PWTd\;\times 1\;+PWTd\right)-2\;LVIDd}$

LV systolic wall tension (dynes) = $\frac{1.333\;\times SBP\;\times LViDs}{2}$

## Statistical analysis

SPSS version 11.0 (SPSS, Inc, Chicago Illinois) was used for statistical analysis. Continuous variables were expressed as mean ± SD and categorical variables as percentages. We assessed differences in categorical variables by Chi-square analysis while the unpaired *t*-test was used for comparison of continuous variables between men and women.

To determine the independent predictors of LV mass, multiple regression models were created. Univariate regression was first performed for each continuous and categorical variable. Thereafter variables having a significant correlation with LVM were entered in stepwise multiple regression models.

In the final model, variables that exhibited a high degree of collinearity were added or deleted iteratively. This was to achieve a model with consistency and conciseness. The probability value to enter and remove was set at *p* ≤ 0.05.

## Results

Of the 285 subjects enrolled, there were 153 men (53.7%) and 132 women (46.3%); overall mean age was 58.2 ± 13 years. Of these, 146 patients were presenting for the first time at the hypertension clinic (51.2%).

About 70.2% of the subjects were on thiazide diuretics and 56% were on calcium channel blockers. Angiotensin receptor blockers and beta-blockers were prescribed for 44.6 and 13.7% of the subjects, respectively. More than 60% of the subjects were receiving two or more classes of drugs.

The clinical and demographic characteristics of the subjects are shown in Table 1. There was no significant difference in the mean age between the genders. The men were taller than the women (*p* = 0.0006) while the women were heavier than the men (BMI, 27.2 ± 5.6 vs 25.6 ± 4.8 for women and men, respectively. About 64% of the women were overweight compared to 51% of the men.

**Table 1.** Baseline Characteristic Of 285 Hypertensive Subjects Seen At UCH, Ibadan, According To Gender

*Parameter*	*All subjects (n = 285)*	*Women (n = 132)*	*Men (n = 153)*	*p-value*
Age (years)	58.2 ± 13.7	57.6 ± 13.2	58.8 ± 14.1	0.468
Body weight (kg)	69.7 ± 13.5	70.3 ± 15.1	69.1 ± 11.9	0.436
Height (cm)	162.8 ± 8.7	161.0 ± 8.5	164.5 ± 8.6	0.0006
BMI (kg/m^2^)	26.3 ± 5.0	27.2 ± 5.6	25.6 ± 4.8	0.00081
BSA (m^2^)	1.74 ± 0.18	1.74 ± 0.19	1.75 ± 0.62	0.428
SBP (mmHg)	153.2 ± 29.5	150.7 ± 24.3	155.3 ± 33.2	0.193
DBP (mmHg)	94.2 ± 18.0	92.0 ± 14.5	96.0 ± 20.5	
Pulse press (mmHg)	59.0 ± 21.9	58.7 ± 19.8	59.3 ± 23.5	0.815
MAP (mmHg)	113.8 ± 20.0	111.6 ± 15.8	115.8 ± 22.8	0.080
Heart rate (beats/min)	80.3 ± 19.2	82.2 ± 18.4	78.3 ± 19.8	0.133
Duration of hypertension (range in years)	4.0 ± 7.4 (0–30)	2.55 ± 5.9 (0–30)	5.19 ± 8.20 (0–30)	0.004
Alcohol consumers	70	25	45	0.028
Cigarette smokers	31	11	20	0.138
Diabetes mellitus	17	8	9	0.572
Family history of hypertension	43	17	30	0.085
Overweight/obese (%)	159 (55.8)	84 (63.6)	77 (51)	0.035
Married (%)	249 (87.4)	111 (84.1)	138 (90.2)	0.162

BMI = body mass index, BSA = body surface area, SBP = systolic blood pressure, DBP = diastolic blood pressure, MAP = mean arterial blood pressure.

The duration of hypertension was longer in the men than the women. A history of alcohol consumption was also commoner in men. A positive family history of hypertension was obtained in 43 subjects and more often in men. There was no significant difference in blood pressure, heart rate, history of cigarette smoking, presence of diabetes mellitus, marital status and origin of subjects between the two genders. Table 2 depicts the biochemical characteristics of the subjects.

**Table 2.** The Biochemical Parameters Of Hypertensive Subjects Seen At UCH, Ibadan, According To Gender

*Parameter*	*All subjects*	*Women*	*Men*	*p-value*
Sodium (mmol/l)	138.4 ± 6.4	138.8 ± 5.0	138.1 ± 7.1	0.488
Potassium (mmol/l)	3.77 ± 0.67	3.65 ± 0.63	3.78 ± 0.59	0.202
Creatinine (mg/dl)	1.57 ± 1.31	1.26 ± 0.32	1.79 ± 1.67	0.0064
Uric acid (mg/dl)	7.4 ± 2.9	6.63 ± 2.27	7.91 ± 3.14	0.066
Fasting blood glucose (mg/dl)	111.1 ± 49.4	111.3 ± 38.5	110.9 ± 57.1	0.962
2-hour postprandial glucose (mg/dl)	135.2 ± 74.4	129.6 ± 55.3	140.3 ± 88.7	0.489
Total cholesterol (mg/dl)	190.7 ± 52.3	195.6 ± 43.5	186.9 ± 58.2	0.391
TG (mg/dl)	82.6 ± 7.9	113.5 ± 79.2	117.8 ± 85.7	0.782
HDL (mg/dl)	43.7 ± 20.0	47.02 ± 23.4	41.2 ± 20.8	0.180
LDL (mg/dl)	124.6 ± 50.0	128.6 ± 45.8	121.5 ± 53.1	0.470

TG = triglycerides, HDL = high-density lipoprotein cholesterol, LDL = low density lipoprotein cholesterol.

## Echocardiographic findings

Table 3 and 4 summarise the echocardiographic parameters. Except for septal wall thickness and indices of LV systolic function (ejection fraction and fractional shortening), which where higher in men, all the parameters were similar in the men and women.

**Table 3.** The Echocardiographic Parameters Of 285 Hypertensive Subjects Seen At UCH, Ibadan

*Parameter*	*All subjects*	*Women*	*Men*	*p-value*
Aortic root diameter (cm)	2.87 ± 0.43	2.84 ± 0.40	2.89 ± 0.46	0.354
LA (cm)	3.52 ± 0.72	3.48 ± 0.67	3.56 ± 0.76	0.378
Septum (cm)	1.07 ± 0.28	1.03 ± 0.207	1.11 ± 0.33	0.013
PWTd	0.997 ± 0.22	0.996 ± 0.211	0.998 ± 0.228	0.947
LVIDd	4.86 ± 0.95	4.84 ± 0.91	4.87 ± 0.99	0.817
LVIDs	3.34 ± 0.96	3.39 ± 0.93	3.30 ± 0.99	0.457
E-velocity	0.59 ± 0.22	1.123 ± 0.76	1.03 ± 0.70	0.757
A-velocity	0.61 ± 0.19	58.7 ± 19.8	59.3 ± 23.5	0.0806
E/A ratio	1.07 ± 0.58	1.02 ± 0.46	1.11 ± 6.65	0.190
Deceleration time	199.7 ± 60.8	196.1 ± 61.3	202.5 ± 60.5	0.426

LA = left atrial diameter, PWTd = posterior wall thickness in diastole, LVIDd = LV internal diameter in diastole, LVIDs = LV internal diameter in systole, E = early left ventricular filling phase, A = late left ventricular filling phase.

**Table 4.** The Echocardiographically Derived Parameters In 285 Hypertensive Men And Women Seen At UCH, Ibadan

*Parameter*	*All subjects*	*Women*	*Men*	p*- value*
LVM	188.7 ± 82.4	180.7 ± 67.0	195.7 ± 93.3	0.124
LVM/height^2.7^	50.8 ± 22.1	49.6 ± 16.6	51.9 ± 26.0	0.394
LVM/BSA	116.0 ± 49.9	104.2 ± 37.3	112.5 ± 53.8	0.141
LVM/hypertension	108.6 ± 46.9	111.7 ± 39.1	119.8 ± 57.6	0.172
Relative wall thickness	0.43 ± 0.13	0.428 ± 0.13	0.428 ± 0.14	0.985
LVH/no LVH	111/174	58/74	53/100	0.145
Fractional shortening (%)	32.0 ± 9.2	30.7 ± 9.96	33.0 ± 8.75	0.033
Ejection fraction (%)	58.9 ± 13.4	57.0 ± 14.2	60.5 ± 12.6	0.027
End-diastolic volume (ml)	116.6 ± 54.9	115.3 ± 50.6	117.7 ± 58.4	0.711
End-systolic volume (ml)	51.5 ± 38.7	52.6 ± 36.4	50.5 ± 40.6	0.651
Stroke volume (ml)	65.2 ± 25.7	62.7 ± 25.2	67.2 ± 26.1	0.140
Stroke index (ml/m^2^)	1.74 ± 0.18	1.735 ± 0.191	1.751 ± 0.162	0.428
Cardiac output (ml)	5275 ± 2636	5192 ± 2606	5348 ± 2669	0.623
Cardiac index (ml/m^2^)	3.04 ± 1.50	300.94 ± 1472.2	3072.8 ± 152.40	0.725
Total peripheral resistance (dynes/ml)	0.027 ± 0.015	0.027 ± 0.16	0.026 ± 0.013	0.497
Pulse pressure–stroke volume ratio	1.07 ± 0.73	1.13 ± 0.75	1.03 ± 0.70	0.247
Wall stress (dynes/cm^2^)	338.6 ± 119.9	335.9 ± 106.0	340.9 ± 131.0	0.724
Wall tension (dynes/cm^2^)	186.5 ± 75.9	181.7 ± 68.3	190.7 ± 82.0	0.319

LVM = left ventricular mass, LVH = left ventricular hypertrophy, BSA = body surface area.

When the subjects were grouped into those with increased LV mass (left ventricular hypertrophy) and those with normal LV mass, significant differences were found in their clinical, biochemical and echocardiographic characteristics. Hypertensive subjects with left ventricular hypertrophy were found to be older, heavier and had higher baseline systolic, diastolic and mean arterial blood pressures. Their resting heart rate was also significantly higher. They had higher serum sodium, lower serum potassium (0.015), higher serum creatinine and higher uric acid levels. This group also had higher fasting blood glucose and lipid profiles (total cholesterol, triglycerides and LDL cholesterol). Accordingly they also had lower mean HDL cholesterol levels.

Comparison of their echocardiographic variables revealed that subjects with increased LV mass (LVH) had larger aortic root diameters, left atrial diameters and LV wall thickness. LV internal dimensions (both in systole and diastole), LV enddiastolic and end-systolic volumes, stroke volume and cardiac output were also higher in the group with LVH.

On the other hand, subjects with increased LVM had lower indices of LV systolic function (ejection fraction and fractional shortening), relative wall thickness, total peripheral resistance and pulse pressure–stroke volume ratio. These were all statistically significant. There was no difference in the indices of LV diastolic function (E-velocity, A-velocity, E/A ratio and deceleration time).

## Determinants of LV mass

As shown in Table 5, LV mass correlated fairly well with duration of hypertension (*r* = 0.123), alcohol consumption (*r* = 0.209), cigarette smoking (*r* = 0.190), presence of diabetes mellitus (*r* = 0.187), body weight (*r* = 187) and height (*r* = 0.195). LV mass was also positively related with body surface area (*r* = 0.228), systolic BP (*r* = 0.200) diastolic BP (*r* = 0.235) and mean arterial BP (*r* = 0. 239). Serum creatinine (*r* = 0.225), serum uric acid (*r* = 0.362), aortic root diameter (*r* = 0.238), left atrial diameter (*r* = 0.514), LV end-diastolic volume (*r* = 0.727) and LV end-systolic volume (*r* = 0.989) were all positively and directly related to LV mass.

**Table 5.** Correlation Of LV Mass With Clinical, Biochemical And Other Echocardiographic Variables In 285 Hypertensive Subjects Seen At UCH, Ibadan

*Parameter*	*r*	*r^2^*	p*-value*
Age (years)	0.061	0.004	0.302
Gender (male vs female)*	0.091	0.008	0.124
Duration (years)	0.123	0.015	0.046
Alcohol consumption (yes/no)*	0.209	0.044	0.0004
Cigarette smoking (yes/no)*	0.190	0.36	0.0012
Family history of hypertension (yes/no)*	0.074	0.005	0.214
Diabetes mellitus (yes/no)*	0.139	0.019	0.0185
Weight (kg)	0.187	0.035	0.0016
Height (cm)	0.195	0.038	0.0010
Body mass index (kg/m^2^)	0.065	0.004	0.280
Body surface area (m^2^)	0.228	0.052	0.001
Systolic blood pressure (mmHg)	0.200	0.040	0.0007
Diastolic blood pressure (mmHg)	0.235	0.050	< 0.0001
Pulse pressure (mmHg)	0.076	0.006	0.203
Mean arterial pressure (mmHg)	0.239	0.057	< 0.0001
Sodium (mmol/l)	0.017	0.0002	0.831
Potassium (mmol/l)	–0.123	0.015	0.118
Creatinine (mg/dl)	0.225	0.051	0.002
Uric acid (mg/dl)	0.362	0.131	0.0002
Fasting blood glucose (mg/dl)	0.071	0.005	0.420
2-hour postprandial glucose (mg/dl)	0.046	0.002	0.658
Total cholesterol (mg/dl)	0.121	0.015	0.189
Triglycerides (mg/dl)	–0.013	0.0001	0.886
High-density lipoprotein (mg/dl)	–0.063	0.004	0.516
Low-density lipoprotein (mg/dl)	0.109	0.012	0.268
Heart rate (beats/min)	0.092	0.009	0.123
Aortic root diameter (cm)	0.238	0.056	< 0.0001
Left atrial diameter (cm)	0.514	0.264	< 0.0001
Septal wall thickness (cm)	0.588	0.346	< 0.0001
LV posterior wall thickness (cm)	0.426	0.182	< 0.0001
LV end-diastolic diameter (cm)	0.718	0.516	< 0.0001
LV end-systolic diameter (cm)	0.694	0.482	< 0.0001
Relative wall thickness (cm)	–0.126	0.016	0.034
Fractional shortening (%)	–0.380	0.144	< 0.0001
Ejection fraction (%)	–0.415	0.172	< 0.0001
End-diastolic volume (ml)	0.727	0.528	< 0.0001
End-systolic volume (ml)	0.989	0.471	< 0.0001
Stroke volume (ml)	0.518	0.269	< 0.0001
Stroke index (ml/m^2^)	0.228	0.052	0.0001
Cardiac output (ml)	0.458	0.210	< 0.0001
Cardiac index (ml/m^2^)	0.414	0.171	< 0.0001
Total peripheral resistance	–0.329	0.108	< 0.0001
Pulse pressure–stroke volume ratio	–0.284	0.018	< 0.0001
Wall stress (dynes/cm^2^)	0.670	0.449	< 0.0001
Wall tension	0.255	0.065	< 0.005
Mitral early velocity (E)	0.089	0.008	0.168
Mitral late velocity(A)	–0.050	0.002	0.443
E/A ratio	0.110	0.012	0.090
Deceleration time	–0.072	0.005	0.279

*Values were entered as dummy variables.

All the haemodynamic parameters also showed a relationship with LV mass. The relationship was mostly positive except for fractional shortening (*r* = –0.380), ejection fraction (*r* = –0.415), total peripheral resistance (*r* = –0.329) and pulse pressure/stroke volume ratio (*r* = –0.284), which showed a negative relationship with LV mass. LVM was not significantly related to indices of LV diastolic function. However the relationship with A-velocity and deceleration time was negative

In a stepwise multivariate linear regression analysis, the independent predictors of LV mass were LV wall tension, left atrial diameter, LV wall stress, LV end-diastolic volume, diastolic blood pressure, family history of hypertension, and alcohol consumption. These variables explained about 95% of the variability in LV mass (Table 6). A sub-analysis of the newly presenting hypertensive subjects did not yield additional or different information.

**Table 6.** Independent Predictors Of LV Mass In Hypertensive Subjects Seen At UCH, Ibadan

*Variable*	*b*	*SE*	*Beta*	*p-value*
Wall tension	0.277	0.0	0.324	0.000
Left atrial diameter	17.55	5.74	0.131	0.004
Wall stress	–0.794	0.067	–0.691	0.000
LV end-diastolic volume	1.465	0.118	0.941	0.000
Diastolic BP	0.557	0.215	0.122	0.012
Family history of hypertension	18.10	7.87	0.082	0.026

r^2^ = 0.952 (adjusted 0.945), *F* = 138.77, *p* = 0.000.

## Discussion

Left ventricular mass is a powerful and independent risk factor for cardiovascular events. In this study, the prevalence of echocardiographically defined LVH in subjects aged 20 to 94 years was 38.9%. Previous studies have reported prevalences ranging from 15 to 64%.^24^-^26^

In a study of 50 untreated hypertensive and 50 normotensive subjects, Ganau *et al*.^27^,^28^ showed that systolic BP, stroke–volume index and end-systolic stress/volume index ratio were strong determinants of indexed LVM. Another study by Jones and colleagues^29^ also observed that changes in haemodynamic load may induce LVH and abnormal LV remodelling in hypertension.

These parameters were determined in the present study, where it was found that LVM correlated with end-diastolic volume, end-systolic volume, stroke volume, stroke index, cardiac output and cardiac index. LVM was also found in this study to be related to total peripheral resistance, pulse pressure/stroke volume ratio, LV wall tension and LV wall stress.

Dahan and colleagues^30^ reported LV end-diastolic volume and end-systolic meridional stress-to-volume ratio as the main determinants of LVM in a population of patients on haemodialysis. A recent comprehensive report by Chen and co-workers^31^ is also in keeping with our findings. Major predictors of LVM in our model included LV wall tension, left atrial size, LV wall stress, LV end-diastolic diameter, diastolic BP, family history of hypertension and alcohol consumption.

Parameters that cause the left ventricle to remodel can be categorised as those that affect the external or internal LV loads. To date, it is still unclear how many vascular parameters affect cardiac load. The vascular or haemodynamic parameters may affect blood pressure, properties of the arterial wall, or size of the arteries. For example, systolic blood pressure imposes external load on the left ventricle. It is a reflection of the ejection force of the heart and is influenced by factors such as arterial wall stiffness and transmission speed.

On the other hand, diastolic blood pressure determines the LV end-diastolic pressure, which is the pressure required for the aortic valve to open. It affects the internal load of the left ventricle. In our model, diastolic blood pressure was found to be an independent determinant of LV mass. Its role as a predictor of LVM stems from the fact that stretch on the cardiac myocytes is a stimulant for cardiac hypertrophy. It causes activation of intra-cellular messengers, changes in ionic homeostasis, and increased synthesis of various proteins as well as growth factors.^32^ All these can result in cardiac muscle hypertrophy. The same applies to the role of LV wall tension and wall stress as a predictor of LV mass.

The association between alcohol consumption and LV mass is not well established. A previous report on a Caucasians population has shown that heavy drinking has a positive but weak association with LV mass.^33^ On the contrary, Sax *et al*.^34^ did not observe any relationship between alcohol consumption and LVM.

Our finding that a family history of hypertension is a strong predictor of LV mass corroborates the report of other workers. It has been demonstrated that LV mass is significantly elevated in normotensive offspring of hypertensive parents, and siblings of people with LVH.^35^ Moreover, studies on twins have shown some influence of genetics on LV mass, which was independent of other factors.^36^ This may not be unconnected with higher ambulatory blood pressure loads.

## Negative observations

We did not observe the independent relationship between body mass index and LVM previously reported by other workers.^37^,^38^ BMI showed a weak correlation with LVM in the univariate regression analysis. On the other hand, there was a strong association between BSA and LV mass in this study as reported by some workers, but not an independent determinant.

This study did not find age as an independent determinant of LVM as previously reported.^24^,^39^ Its correlation with LV mass was also weak in the univariate analysis (*r* = 0.061).

There was little gender difference in the echocardiographic parameters. Gender also correlated weakly with LV mass. Two possible reasons are that (1) the influence of gender on LV mass could have been accounted for by other factors such as height, weight and body surface area; (2) the choice of a non-gender-specific partition value for the definition of LVH in this study could have obliterated any gender difference. Some workers have reported gender as a determinant of LV mass.^40^,^41^

Aortic root diameter did not enter our model as a predictor of LVM. The study by Chan *et al*.^31^ has reported aortic root diameter as one of the five strongest determinants of LV mass in their subjects, apart from stroke volume, systolic BP, BMI and end-systolic meridional stress-to-volume ratio.

Although total peripheral resistance showed a strong relationship with LVM in the univariate analysis, it was not an independent predictor of LVM. This corroborates some studies that showed that vascular resistance is not a good index of haemodynamic loading.^42^ We also confirmed the rather surprising, inappropriate and negative relationship between LV mass and indexes of arterial stiffness noted earlier by Chan *et al*.^31^

## Limitations of the study

The first limitation of the study was the fact that it was retrospective and cross-sectional. It can only claim association but not causality. Secondly, clinic or casual blood pressure was measured in the subjects. Many studies have shown that ambulatory blood pressure is more closely associated with LV mass than clinic blood pressure.

Thirdly, other variables such as insulin resistance, blood viscosity, and carotid intima–media thickness that have been documented as determinants of LV mass were not assessed in this study. Fourthly, this study did not correlate ECG findings with ECHO findings. The authors will explore this in future research.

Lastly, there may be concern about the independence of the haemodynamic parameters, which were derived from some M-mode measurements also used in the calculation of LV mass. This concern has been addressed in the recent report by Chan *et al.*31 They tested the reliability of M-mode parameters by comparing them with those obtained with the two-dimensional echocardiographic truncated ellipsoid formula, which were devoid of M-mode-derived parameters in 1 238 subjects. They noted a lower overall model variance (0.59 vs 0.74) but with the same top predictors of LV mass contributing to 98% of the total variance.

## Conclusion

This study found that the most important determinants of LV mass were LV wall tension, left atrial size, LV wall stress, diastolic BP, a family history of hypertension, and alcohol consumption. These factors appeared to explain about 95% of the variability in LV mass in hypertensive Nigerians seen at the University College Hospital, Ibadan. Mechanical or haemodynamic factors, possibly interacting with genetic and social factors are the likely determinants of LV mass in Nigerian hypertensive men and women. Therefore modulation of these factors pharmacologically or non-pharmacologically will be of benefit in the management and control of hypertension in this population.

The implications of this study are that measures that effectively modulate wall stress and wall tension as well as blood pressure control, for example antihypertensive therapy, can cause early regression of LVH. Early screening of relatives of hypertensive subjects may provide an effective blood pressure-control programme. Non-pharmacological treatment such as avoidance of excessive alcohol consumption may be an effective tool in a blood pressure-control programme.

However, based on the recent findings by the LIFE study,^10^-^12^ which showed a correlation between ECG and ECHO findings, as well as the recommendation of the World Heart Organisation, ‘echocardiography in hypertensive patients in sub-Saharan Africa should be used only for research purposes and is not cost effective in resource-poor countries’.
